# Tracking Microplastics Contamination in Drinking Water Supply Chain in Haikou, China: From Source to Household Taps

**DOI:** 10.3390/toxics12110793

**Published:** 2024-10-30

**Authors:** Xiangxiang Li, Yihan Yu, Mei Yang, Shaobai Wen, Jun Zhang

**Affiliations:** 1 NHC Key Laboratory of Tropical Disease Control, School of Tropical Medicine, Hainan Medical University, Haikou 571199, China; lxx@hainmc.edu.cn (X.L.); myang_1995@163.com (M.Y.); 2 School of Public Health, Hainan Medical University, Haikou 571199, China; 3 School of Stomatology, Hainan Medical University, Haikou 571199, China; yuyihan20042024@163.com

**Keywords:** drinking water, microplastics, risk assessment

## Abstract

The presence of microplastics (MPs) in aquatic environments has become a significant global concern due to their potential adverse effects on human health. This study aimed to investigate the contamination of MPs throughout the drinking water supply chain in Haikou City, China, and to conduct risk assessments regarding the relationship between MPs contamination and human health. The results revealed that the abundance of MPs in raw, treated, and tap water was 0.6 ± 0.6, 5.2 ± 2.7, and 1.2 ± 1.1 particles·L^−1^, respectively. Fragments were identified as the most prevalent shape across all samples, with the size category of 20–50 μm showing the highest abundance of MPs. Among the 11 types of polymers identified, polyethylene and polypropylene accounted for 50% and 29%, respectively. The potential risk index values were significantly higher for treated water (370.26) and tap water (303.85) compared to raw water (13.46), suggesting that plastic pipes may be a key contributor to MPs contamination in drinking water. Therefore, efforts should be directed toward developing pipes with low release rates of MPs, as well as improving detection methods for smaller particles and accurately assessing associated risks.

## 1. Introduction

Plastic is widely used in daily life due to its stability, lightweight nature, durability, and low cost [1]. In 2020, global plastic production reached 360 million tons, generating 29 million tons of waste of which only 21% was treated [2]. Improper disposal has led to significant pollution from microplastics (MPs), which are defined as particles from 1 µm to 1 mm or 5 mm [3,4]. MPs are categorized into primary MPs, which are intentionally manufactured for cosmetics and other uses [5], and secondary MPs, which result from the degradation of larger plastics [6,7].

The proportion of MPs in global plastic accumulation is expected to reach 13% by 2060 [8]. MPs are prevalent in various environments, including oceans [9], rivers [10], air [11], soil [12], and food [13]. Consequently, individuals inevitably ingest MPs through multiple pathways, resulting in their accumulation in bodily fluids, tissues, organs, and feces [14]. Furthermore, the presence of MPs in the human body may pose health risks [15].

Drinking water is a primary pathway for MPs to enter the human body, particularly through tap water. Studies have detected MPs in tap water worldwide, with an average concentration of 39 ± 44 particles·L^−1^ (≥19 μm) across sites in Japan, the US, and the European Union [16]. In China, tap water from 38 cities, including Beijing, Guangzhou, and Shanghai, had an average of 440 ± 275 particles ·L^−1^ [17]. Freshwater sources like rivers, lakes, and reservoirs are often used for drinking water [18]. For example, the Yangtze River contained 3738 ± 461 particles·L^−1^ [10], while surface water samples from the Danjiangkou Reservoir showed MPs concentrations ranging from 467 to 15,017 particles·m^−3^ [19]. Drinking water treatment plants (DWTPs) play a vital role in removing MPs and preventing their entry into the human body [20,21,22,23,24]. Despite their effectiveness, MPs have been detected in both treated and tap water, and the extent of their presence in drinking water across various regions remains unclear. Furthermore, research on the occurrence, fate, and transformation of MPs from raw water to tap water is limited, as are risk assessment studies.

Water quality is directly linked to public safety and well-being. This study aimed to investigate the abundance of MPs in raw water, treated water, and household tap water within the drinking water supply chain in Haikou, China. The objectives were as follows: (1) to quantify MPs in drinking water and compare levels across different samples; (2) to characterize the distribution of MPs by shape, size, and material composition; and (3) to assess potential risks associated with MPs in various samples. This research seeks to address knowledge gaps regarding MPs contamination in Haikou’s drinking water and provide valuable insights for improving standards related to MPs content in DWTPs.

## 2. Materials and Methods

### 2.1. Sampling Sites

**Figure 1:**
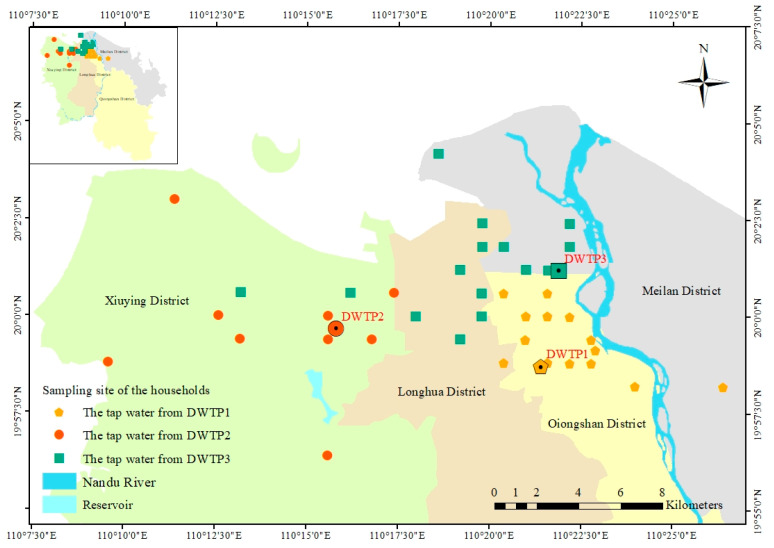
Geographical location and sampling points in Haikou City, Hainan Province.

The main urban area of Haikou city is served by three main drinking water treatment plants (DWTPs), which collectively supply 840,000 m^3^ day^−1^ to approximately 2.5 million residents. Samples of both raw and treated water were collected from DWTP1, DWTP2, and DWTP3 (names withheld for confidentiality). DWTP2 and DWTP3 receive water from the Nandu River, which is the largest river in Hainan Province, spans over 333.8 km, and has a watershed area of 7033 km^2^. DWTP1 sources its water from a reservoir. Additionally, drinking water samples were collected from 46 household taps across various user types, including institutions, schools, children’s venues, and medical facilities. All sampling points (raw, treated, and tap water) were located within control sections established by the Haikou Center for Disease Control and Prevention (Figure 1).

### 2.2. Preparation of Water Samples

Sampling was conducted from 11 to 13 September 2023 under warm, sunny conditions. Raw water was collected at the DWTPs’ inlet, treated water at the outlet [23,25], and tap water from a residential tap, after allowing the water to run for 10 min [18]. Three replicate samples were taken at each site [5]. A total of 5 L from each site was concentrated using a stainless-steel sieve with a 30 μm opening. Residuals were rinsed into a clean 250 mL glass bottle. To prevent contamination, the sieve and bottles were washed before use. Water samples were filtered through a 0.45 μm mixed cellulose esters (MCE) membrane (Xingya Purification Material Factory, Shanghai, China) using vacuum filtration on a laminar flow table. Residuals in the glass bottles and filters were rinsed with ultrapure water, and the filter membranes were stored in a closed glass dish for subsequent analysis. The whole process is illustrated in Figure S1.

### 2.3. Identification of MPs with µ-Raman Spectroscopy 

After sample preparation, the MCE membrane was divided into columns for MPs observation using a confocal Raman spectroscopy microscope (inVia™ InSpect, Renishaw, Wotton-under-Edge, UK) with various objectives (5×, 20×, and 50×). Selected MPs were further analyzed by field emission scanning electron microscopy (FE-SEM, ZEISS, Jena, Germany). Qualitative analysis was conducted using a Raman instrument (532 nm laser, 10 s exposure, 50× objective, 4.2 mW, 1800 mm^−1^ grating, and 200–3200 cm^−1^ shift). The spectrometer was calibrated to the 520.5 cm^−1^ line of an internal silicon prior to spectral acquisition. All raw Raman spectra were submitted to baseline correction by WiRE software 5.6 (Renishaw, Wotton-under-Edge, UK) to decrease noise and improve spectrum quality. The final Raman spectra collected for each MP were compared with an in-house specific spectral library (Renishaw Polymeric Materials Database r03-S/N H12345). A Raman spectrometer was employed to capture Raman signals from particles, with the resulting data imported into specialized library software. This software automatically analyzed the characteristic peaks and peak intensities of the particle spectra using a classical algorithm. It then compared these spectra with a database, generating a series of spectral candidates and their corresponding Hit Quality Index (HQI) values. The value of a HQI more than 0.70 was determined [26] or characteristic peaks were compared [5]. Particle dimensions were measured with Image-Pro Plus 6.0 software (Media Cybernetics, Rockville, MD, USA) and categorized into four size groups: 1–20 μm, 20–50 μm, 50–100 μm, and >100 μm. Morphologically, they were classified as fragments, fibers, or spheres. Fibers were elongated, spheres were circular, and particles not fitting these categories were labeled as fragments, with size measured between the two most distant points [27].

### 2.4. Quality Assurance and Control

Parallel blank experiments (*n* = 3) detected 3 MPs, correcting final statistical results. Glassware was washed three times with ultrapure water and dried before use (manufactured by Jinghong Experimental Equipment Company, Shanghai, China). Fiber and plastic use were minimized, and the operator wore a cotton lab coat, mask, and non-plastic gloves in a clean environment. Sample processing occurred on a laminar flow bench, regularly cleaned with 75% ethanol.

### 2.5. Potential Risk Assessment of MPs

There is limited direct evidence linking MPs to significant health risks for humans, but their potential threats remain a concern. A risk assessment model has been applied to evaluate MP hazards in various contexts [28], including atmospheric assessments [9] and drinking water analysis [18,29] as detailed below:

(1) $$RI=\sum_{i=1}^{n}E_{i}$$

(2) $$E_{i}=T_{i}\times \left(\frac{C_{i}}{C_{o}}\right)$$

In this study, *RI* is the potential risk index, and *E_i_* is the potential single risk index. *T_i_* denotes the chemical toxicity coefficient for polymers [30], while *C_i_* is the observed abundance of MPs. Lacking ideal baseline concentrations for raw, treated, and tap water, we used the global average of 5.45 particles·L^−1^ in tap water [13] as a reference. A standard *RI* grade [29] was used to calculate *E_i_* for the analyzed water samples.

### 2.6. The Estimated Daily Intake (EDI) of MPs

The impact of MPs on human health varies with consumption quantity. This study assessed human ingestion through drinking water using the estimated daily intake (*EDI*) of MPs [27] not calculated as follows:

*EDI (MPs/Kg/d) = (C × IR)/BW* (3)

Here, *C* is the concentration of MPs in drinking water (particles·L^−1^), and ingestion rate (*IR*) is the daily water volume consumed. Established *IR* is 1.85 L·d^−1^ for adults (>18 years), 1.12 L·d^−1^ for children (3–18 years), and 0.60 L·d^−1^ for infants (0–3 years), with body weights (*BW*) of 60.6 kg, 27.25 kg, and 9.65 kg, respectively [31].

### 2.7. Statistical Analysis

The data were expressed with the mean ± standard deviation (SD). Significant differences were obtained by one-way analysis of variance (ANOVA) and Tukey’s honestly significant difference (HSD) post hoc test (*p* < 0.05).

## 3. Results and Discussion

### 3.1. The Abundance of MPs

**Figure 2:**
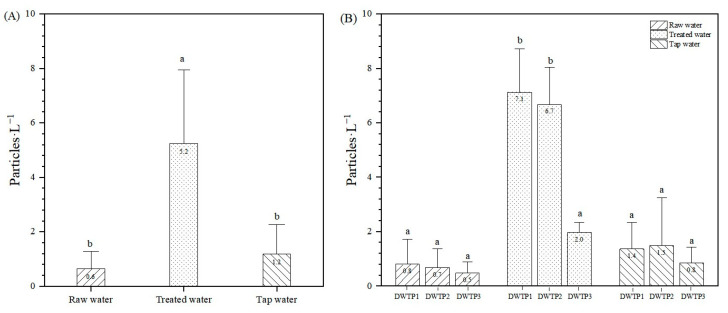
The abundance of MPs in samples. (**A**) The abundance of MPs in raw water, treated water and tap water; (**B**) the abundance of MPs in raw water, treated water, and tap water at DWTP1, DWTP2, and DWTP3. Values with the same superscript letters between groups are of no significant difference (*p* > 0.05); those with different letters are of significant difference (*p* < 0.05). The data were expressed with the mean ± SD, *n* = 3.

A total of 147 treatment samples were analyzed, revealing MPs contamination in 130 samples, indicating an 88% prevalence. The abundance of MPs was 0.6 ± 0.6 particles·L^−1^ in raw water, 5.2 ± 2.7 particles·L^−1^ in treated water, and 1.2 ± 1.1 particles·L^−1^ in tap water (Figure 2A). Sand filtration can remove most of the MPs in conventional DWTPs. The removal of MPs and synthetic fibers in the main DWTP of Geneva, Switzerland, relies on coagulation-flocculation-sedimentation treatment followed by sand filtration [32]. However, drinking water can also be contaminated with MPs in certain treatment units of DWTPs such as the ozonation unit after sand filtration, which can introduce nanoplastics into drinking water [33]. The increase in MPs post-treatment was likely due to NaClO disinfection, which can degrade polypropylene (PP), polyethylene (PE), and polyvinyl chloride (PVC) [30,34]. The migration of these MPs mainly originated from pipes used within DWTPs such as fittings and tanks [6]. However, the abundance of MPs in tap water was lower than that in treated water; maybe treated water is typically sampled over a specific period, while tap water may sit or flow for a time, which can dilute some MPs. When comparing the average abundance of MPs across different drinking water supply chains, in drinking water supply chain 1, MPs abundance was 0.8, 7.1, and 1.4 particles·L^−1^, indicating an initial increase of 7.9 times followed by a decrease of 0.8 times. Supply chain 2 showed values of 0.7, 6.7, and 1.5 particles·L^−1^, with an increase of 9.0 times and a decrease of 0.8 times. Similarly, supply chain 3 exhibited measurements of 0.5, 2.0, and 0.8 particles·L^−1^, reflecting an increase of 3.2 times followed by a decrease of 0.6 times (Figure 2B). These results suggested that pipeline length affected MPs release [34]. DWTP3 exhibited significantly lower levels of MPs in treated water compared to DWTP1 and DWTP2, likely due to recent renovations. Pipes also contributed to MPs contamination in tap water, with PP, PE, and Polyethylene terephthalate (PET) detected in rural systems like Chongqing, China. Although DWTPs removed PP and PE, PE was found again in tap water [35]. Pipe degradation from aging may increase the release of abrasive particles, contributing to higher MPs levels in treated and tap water compared to raw water [34].

The concentration of MPs in the surface water of the Nandu River ranged from 0.5 to 0.8 particles·L^−1^, which was significantly higher than the previously reported 406 ± 223 particles·m^−3^ [5]. This river, which is located in a forest reserve with minimal industrial activity and a ban on disposable plastics, contrasted sharply with the Yangtze River, which exhibited much higher levels of 6614 ± 1132 and 3738 ± 461 particles·L^−1^ [10,23]. The presence of MPs was influenced by human activities (economy and agriculture) [36], rainfall [20,25,37], and air deposition [18]. Comparative analysis with previous studies was complicated by methodological differences (Table S1). The majority of studies on the detection of MPs in water have utilized FT-IR and Raman spectroscopy [6,10,20,23,24,25,28,34,37]. However, there has been inconsistency in the size ranges of MPs examined. Some studies focused on MPs larger than 1 μm, while others considered only those exceeding 20 μm. In terms of abundance, raw water samples from Tehran, the Czech Republic, and Changsha showed MPs concentrations ranging from 1588 to 2255, from 1473 to 3605, and 2753 particles·L^−1^ for sizes greater than 1 μm, respectively [18,20,23]. In contrast, a study in Zahedan reported significantly lower concentrations from 0.078 to 0.128 particles·L^−1^ for sizes over 5 μm and 20 μm [34]. These discrepancies suggested an underestimation of MPs abundance due to the exclusion of smaller particles, which posed significant health risks, including oxidative stress and inflammation [15]. Future research should establish standardized methodologies for accurately assessing MPs in the environment to facilitate better comparisons across studies.

Fiber and fragment MPs were the predominant forms identified in various studies, consistently showing significant abundance. Common polymers, such as PP and PE, were widely distributed, along with PET and PVC. In Tianjin, China, nylon and polyester (PEST) were the main constituents, with laundry wastewater identified as their primary source [28]. At least ten types of MPs were detected in drinking water [6,20,23,37]. For instance, samples from two DWTPs along the Úhlava River in the Czech Republic revealed 13 distinct types of MPs, predominantly cellulose acetate (CA), PET, PVC, and PE. Additionally, spectral analysis identified artificial additives like dyes and flame retardants, highlighting potential environmental and health risks associated with MPs [23]. In summary, the widespread presence of these materials emphasizes the serious implications of MPs pollution for ecosystems and human health.

### 3.2. Morphology and Size

In this study, MPs exhibited various morphologies, including fibers, spherical particles, and fragments, which were classified by size (1–20 μm, 20–50 μm, 50–100 μm, and >100 μm). Their morphology was analyzed using SEM as illustrated in Figure S2. Fragmented MPs displayed relatively rough surfaces with irregular edges (Figure S2A), while spherical MPs exhibited slightly rough surfaces after being peeled (Figure S2B). Fibers appeared long and slender with uniform diameters (Figure S2C).

The shape, size, and type distributions of MPs in samples from different supply chains are presented in Figure S3. Three morphologies were identified as fragments, fibers, and spheres. Fragments were the predominant morphotype, showing consistent abundance throughout the study period. In contrast, the presence of fibers decreased from raw water to tap water, while spherical particles were found in very limited numbers. Notably, minor film-shaped MPs were detected in tap water from Gauteng, South Africa [27]. Plastic control strategies significantly influenced the occurrence of rare spherical and non-film MPs in our study, including bans on the production and sale of certain PE films for agricultural use, restrictions on single-use plastic bags, and prohibitions on plastic microbeads in daily chemical products in Hainan Province [5]. Particles sized 50–100 μm were prevalent across all samples, but the 20–50 μm size group was the most abundant, comprising 50% of the total sample. Interestingly, MPs ranging from 1 to 20 μm were absent in raw water but were detected in both treated and tap water.

**Figure 3:**
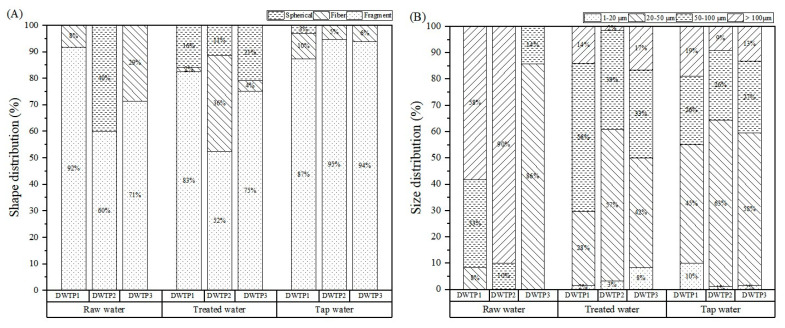
Morphology and size distributions of MPs in samples from different supply chains. (**A**) Morphology distributions of MPs in samples from different supply chains; (**B**) size distributions of MPs in samples from different supply chains. The data were expressed with the mean ± SD, *n* = 3.

As shown in Figure 3A, fragments accounted for a significant proportion of MPs, ranging from 60% to 92% in raw water, from 52% to 83% in treated water, and from 87% to 97% in tap water. These fragments primarily arose from human activities, including littering and various industries like plastics and machinery, and under went weathering and photodegradation. Fibers, resulting from textile production, laundry wastewater, and fishing gear, comprised from 8% to 29% of MPs in raw water, from 2% to 36% of MPs in treated water, and from 6% to 10% of MPs in tap water [38,39,40]. In DWTP2 raw water, spherical MPs represented about 40% of MPs, with none found in tap water. In treated water, they constituted from 11% to 21% of MPs, while tap water from DWTP1 contained approximately 3%. Spherical MPs typically originated from personal care products or the degradation of larger plastics [19].

In raw water, MPs > 100 μm were the most prevalent (17–90%), followed by 50–100 μm (10–33%), and 20–50 μm (8–14%) at DWTP1 and DWTP3. Larger MPs exhibited a higher removal efficiency, with those >100 μm decreasing to 2–17% after treatment. In treated water, the dominant size was 50–100 μm (33–56%), followed by 20–50 μm (28–57%), and 1–20 μm (2–8%). In tap water, MPs > 100 μm represented 9–19%, while 20–50 μm particles accounted for the largest proportion (45–63%), followed by particles sized 50–100 μm (26–27%) and 1–20 μm (1–10%), which was likely due to pipe wear and aging (Figure 3B). The increase in smaller particles indicated that they may arise from the fragmentation of larger MPs through physical, biological, or chemical processes. Microscopic images revealed rough surfaces and scratches on MPs, with small fragments surrounding larger ones (Figure S4), suggesting degradation may produce additional smaller particles [18,41].

### 3.3. Composition of MPs

The Raman spectra of the selected samples, shown in Figure S5, identified 11 types of polymers, including PE, PVC, PP, polystyrene (PS), polyformaldehyde (POM), polymethyl methacrylate (PMMA), and polycarbonate (PC), etc.

**Figure 4:**
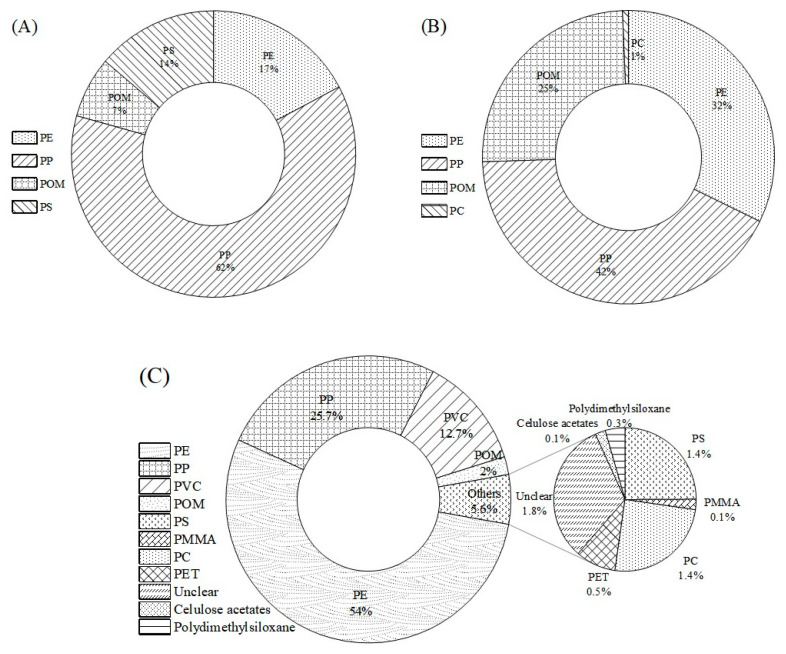
Material composition of MPs in samples. (**A**) Raw water; (**B**) treated water; (**C**) tap water. The data were expressed with the mean ± SD, *n* = 3.

The distribution of polymer types from raw to tap water is illustrated in Figure 4A–C. PP (25.7–62%) and PE (17–54%) were the most prevalent polymers in all samples, reflecting their extensive use in products like plastic bags, agricultural films, and pipes, etc. [16,20,37]. With densities lower than that of water (PP: 0.90–0.91 g/cm^3^; PE: 0.92–0.97 g/cm^3^), these materials tended to float and disperse easily in aquatic environments [24,42]. Notably, POM was detected throughout the drinking water chain (2–25%), which was less common in the existing literature as it is primarily used in automotive and electronic components [43]. Small quantities of spherical and fragmented PS were identified in both raw and tap water, which was consistent with previous research, as PS was often sourced from packaging materials [23,42]. While some polymers were found in both raw and tap water, the composition of MPs varied post-treatment, with PVC, PMMA, and PC appearing in treated water. PC was uniquely detected in this study and is commonly used in automotive and electronic applications, while PVC was primarily employed in pipes [34,44,45]. Conversely, PMMA was rarely detected in tap water, which was consistent with findings from a consumer faucet report in Zaheda. This polymer is commonly used in construction materials, transportation equipment, and medical supplies, among other applications [34]. Overall, the diversity of MPs increased during the conveyance of tap water, indicating that the supply system may also contribute to the presence of MPs in tap water [29].

### 3.4. Risk Assessment

**Figure 5:**
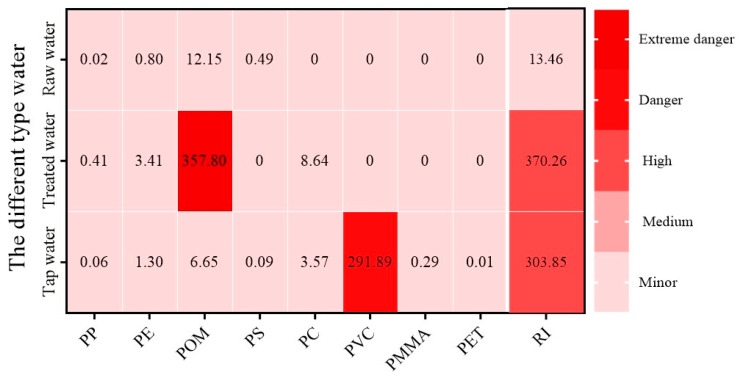
*Ei, RI*, and risk assessment levels in raw, treated, and tap water. The data are based on MPs abundance (mean ± SD), *n* = 3. PS, PMMA, PVC, POM, PP, PE, PC, and PET. (*E_i_* minor: <40; *E_i_* medium: 40–80; *E_i_* high: 80–160; *E_i_* danger: 160–320; *E_i_* extreme danger: ≧320; *RI* minor: <150; *RI* medium: 150–300; *RI* high: 300–600; *RI* danger: 600–1200; and *RI* extreme danger: ≧1200). Cellulose acetates, and polydimethylsiloxane lack toxicity data, and, therefore, their hazard scores cannot be determined.

An initial evaluation of the potential risks associated with MPs in various samples was conducted (Figure 5). The results revealed risk index (*RI*) values of 13.46 for raw water, 370.26 for treated water, and 303.85 for tap water. The highest *RI* value was observed in treated water, indicating a significant increase in the number of MPs present. Both treated and tap water were classified as high risk, with detailed results provided in Tables S2–S4. Overall, there was a general increase in the potential risk of MPs from raw water to tap water. The high environmental impact (*E_i_*) of these polymers was mainly due to their toxicity (*T_i_*). PP and PE had lower hazard levels. In contrast, plastics like POM (1500), PC (1177), PMMA (1021), and PVC (10551) were higher risk and may be carcinogenic [30]. Therefore, it is crucial to focus on highly toxic polymers, even at low concentrations. Clinical evidence suggested a potential connection between MPs exposure and human diseases. For instance, fecal MPs concentrations were significantly higher in patients with inflammatory bowel disease (IBD) compared to healthy individuals, correlating with IBD severity [46]. Additionally, the abundance of PS particles in the villus tissue of patients with unexplained recurrent abortion was higher than that in a healthy control group [47]. Six types of MPs polymers (PET, PMMA, POM, PP, PS, and PVC) were detected in cirrhotic liver tissue at significantly higher levels than in liver samples from individuals without underlying liver disease [48]. A prospective study also found that PE and PVC may increase the risk of myocardial infarction or stroke in individuals with a carotid plaque [49]. These findings underscore the need for further research into the toxic mechanisms of MPs on human health.

### 3.5. The EDI of MPs

The *EDI* of MPs for adults, children, and infants was 0.036, 0.049, and 0.074 MPs/kg/d, respectively (Table S5). Notably, infants exhibited an *EDI* nearly double that of adults, suggesting a decrease in MPs consumption with age. In contrast, the *EDI* in Beijing, China, was significantly higher, measuring 2.68 MPs/kg/d for adults, 3.61 MPs/kg/d for children, and 5.47 MPs/kg/d for infants, highlighting regional differences in MPs abundance [31]. Factors such as *IR* and *BW* play a crucial role in influencing *EDI* results across different ages and locations. In Zahedan, Iran, the *EDI* ranged from 0.0002 to 0.0157 MPs/kg/d for adults and from 0.0004 to 0.0413 MPs/kg/d for children [34]. Drinking water is a key pathway for MPs entering the human body, where they can accumulate and be excreted. Recent research has revealed the presence of MPs in the human body. Notably, PP-MPs were identified in fecal samples from China [14], while fragmented MPs, including PP, PE, PVA, and PVC, were discovered in urine from several cities in southern Italy [50]. Future research should focus on actual exposure levels from drinking water based on quantitative in vitro to in vivo extrapolation and pharmacokinetics modeling, as well as the absorption, excretion, distribution, and accumulation patterns of MPs in the human body [51].

## 4. Conclusions

This study investigated the presence of MPs in the drinking water system of Haikou City, China. The results revealed that MPs were detected in 88% of the collected samples. Treated water showed the highest abundance of MPs (5.2 ± 2.7 particles·L^−1^), followed by tap water (1.2 ± 1.1 particles·L^−1^), while raw water had the lowest abundance. This suggested that the transportation of water through plastic pipelines increased the risk of MPs contamination in tap water. Among the three types of water samples, particles in the size range of 20–50 μm were the most prevalent, with fragmented and fibrous MPs being the dominant shapes. PE made up the largest portion of the MPs at 50%, followed by PP at 30%. Additionally, tap water had a more complex composition, with 10 different types of MPs detected, compared to only 4 types found in the other two water samples. This further underscored the role of pipeline transport in increasing the risk of MPs contamination. The *RI* for MPs in the different water samples indicated that the potential risk levels of treated and tap water fell into a high-risk category. Moving forward, it is essential to conduct large-scale, comprehensive studies on the presence of MPs in drinking water and to develop detection methods for smaller particles or nanoplastics for a more accurate assessment of MP risks in drinking water. These efforts will enhance our understanding and assessment of the risks associated with drinking water quality.

## Data Availability

Data will be made available on request.

## Supplementary Materials

The following supporting information can be downloaded at https://www.mdpi.com/article/10.3390/toxics12110793/s1, Figure S1: Construction work flow of the research; Figure S2: Typical MPs image with different shapes in samples; Figure S3: MPs characteristics in drinking water system; Figure S4: Fragments SEM images of MPs; Figure S5: Partial Raman spectra of the detected MPs from water samples; Table S1: Comparison of the abundance of MPs; Table S2: Potential risk of different MPs in raw water; Table S3: Potential risk of different MPs in treated water; Table S4: Potential risk of different MPs in tap water; Table S5: The *EDI* of MPs ingested by adults, children and infants via drinking water.
