# Supplementary material for: Tracking Microplastics Contamination in Drinking Water Supply Chain in Haikou, China: From Source to Household Taps
Source: Toxics. 2024 Oct 30;12(11):793. doi: 10.3390/toxics12110793 (PMC11597948; doi:10.3390/toxics12110793)
Supplement: Supplementary file 1 [file toxics-12-00793-s001.zip › toxics-3235812-supplementary.pdf]

Supplementary Data

Tracking MPs contamination in drinking water supply chain in Haikou, China: From source to household taps

Xiangxiang Li[1,2], Yihan Yu[3], Mei Yang[1], Shaobai Wen[1*], Jun Zhang[1*]

1 NHC Key Laboratory of Tropical Disease Control, School of Tropical Medicine, Hainan Medical University, Haikou 571199, China; lxx@hainmc.edu.cn (X.L.); myang_1995@163.com (M.Y.)

2 School of Public Health, Hainan Medical University, Haikou 571199, China

3 School of Stomatology, Hainan Medical University, Haikou 571199, China; yuyihan20042024@163.com (Y.Y.)

* Correspondence: hy0211050@hainmc.edu.cn (S.W.); jun_zh1979@163.com (J.Z.)

Contents

Figure S1. Construction work flow of the research.

Figure S2. Typical MPs image with different shapes in samples.

Figure S3. MPs characteristics in drinking water system. (A) MPs characteristics in the raw water; (B) MPs characteristics in the treated water; (C) MPs characteristics in the tap water.

Figure S4. Fragments SEM images of MPs.

Figure S5. Partial Raman spectra of the detected MPs from water samples.

Table S1. Comparison of the abundance of MPs.

Table S2. Potential risk of different MPs in raw water.

Table S3. Potential risk of different MPs in treated water.

Table S4. Potential risk of different MPs in tap water.

Table S5. The EDI of MPs ingested by adults, children and infants via drinking water.

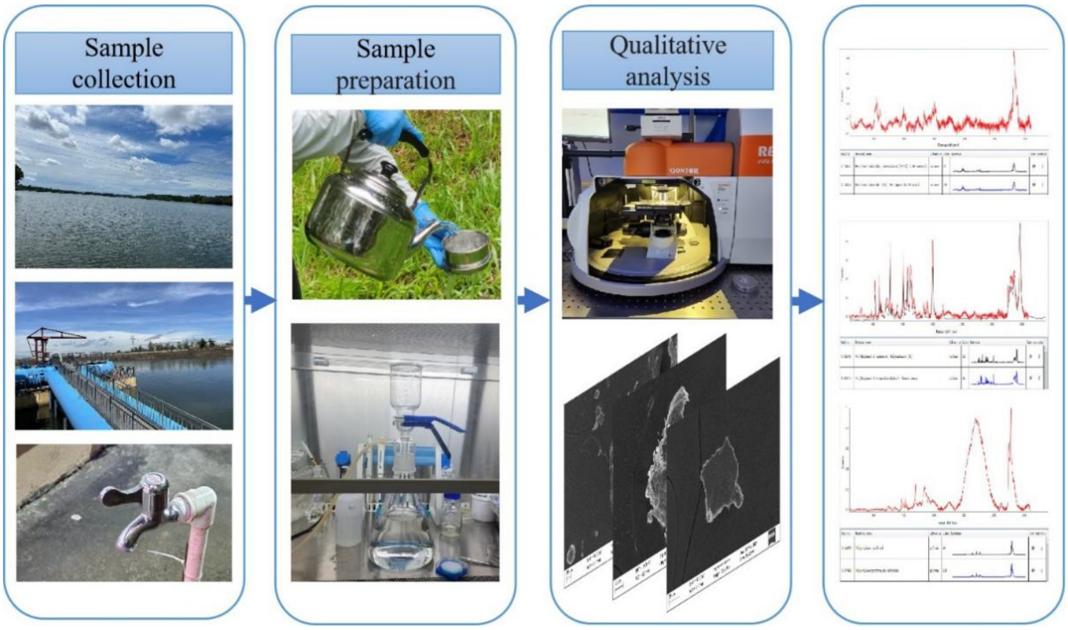

Figure S1. Construction work flow of the research.

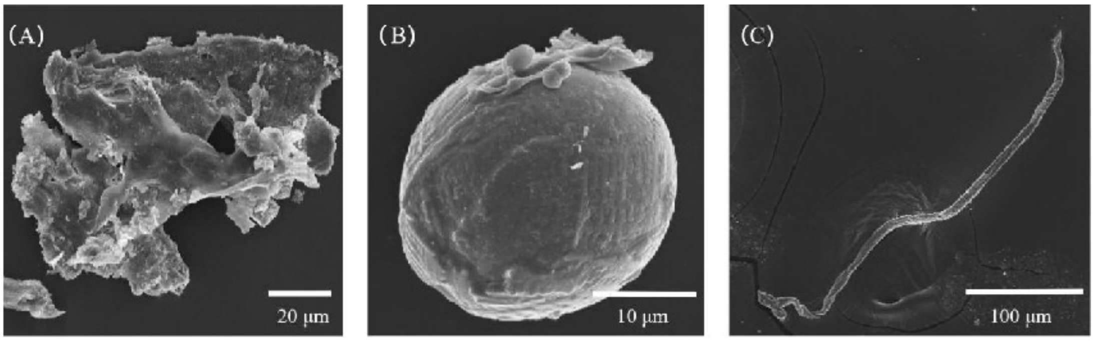

Figure S2. Typical MPs image with different shapes in samples. (**A**) Fragments; (**B**) spherical; (**C**) fibers. Scale bars are provided in the bottom corner of each image.

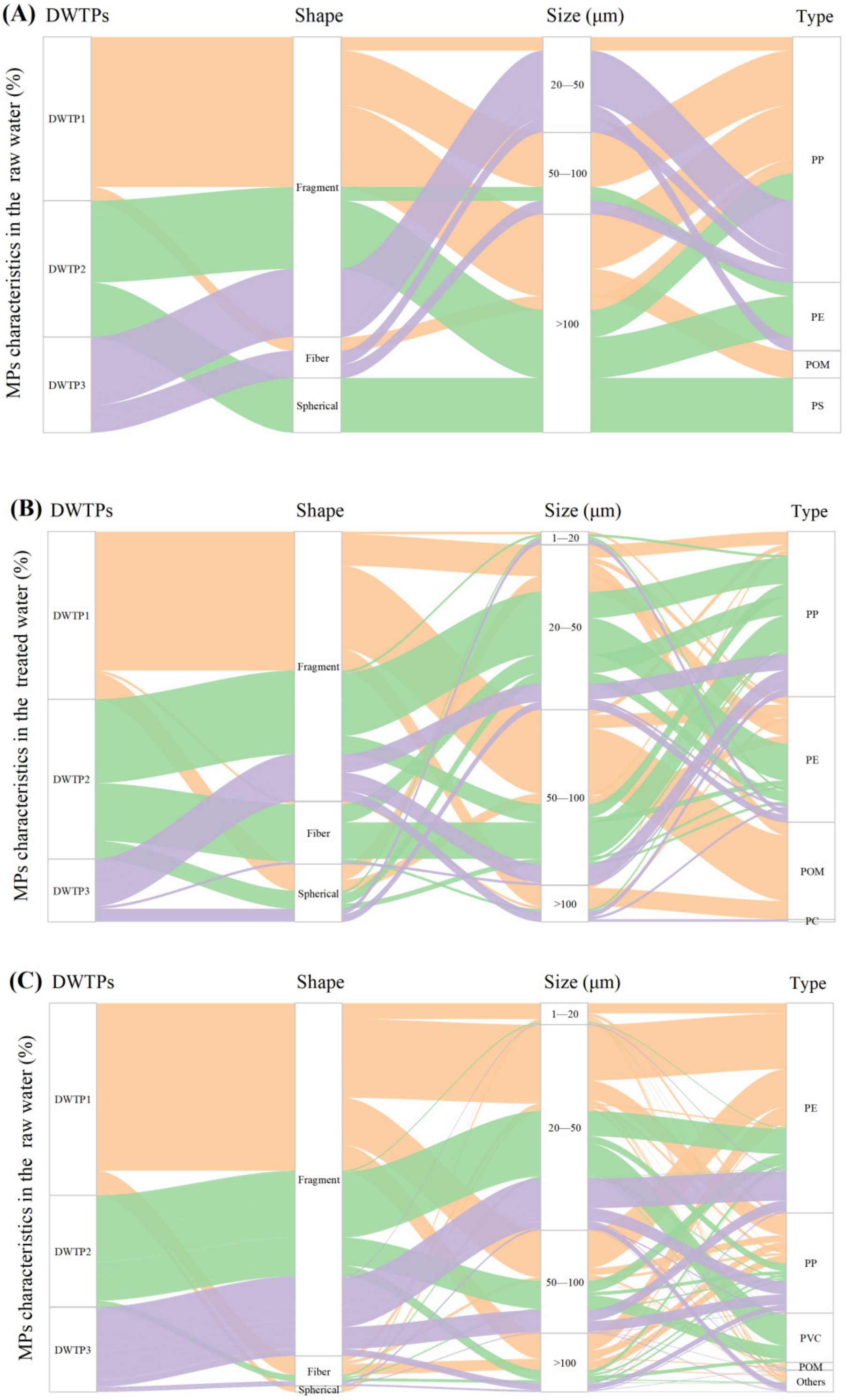

Figure S3. MPs characteristics in drinking water system. (**A**) MPs characteristics in the raw water; (**B**) MPs characteristics in the treated water; (**C**) MPs characteristics in the tap water. Other: PC, PS, PET, PMMA, Polydimethylsiloxane, Cellulose acetates, Unclear (materials individually comprising <2%).

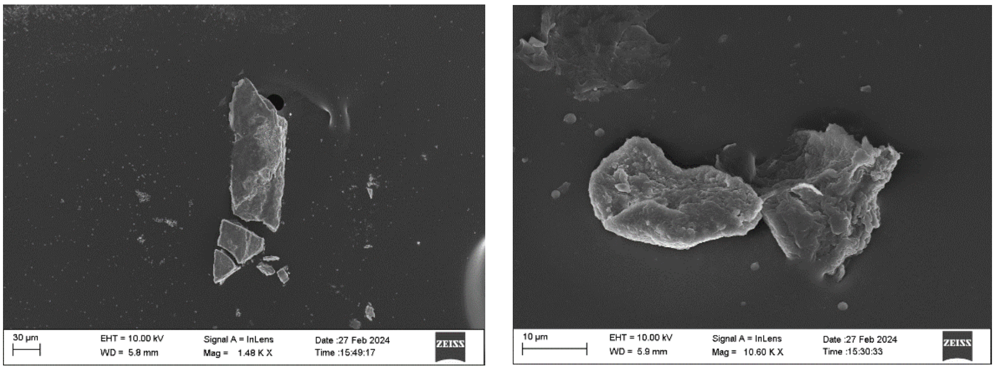

Figure S4. Fragments SEM images of MPs.

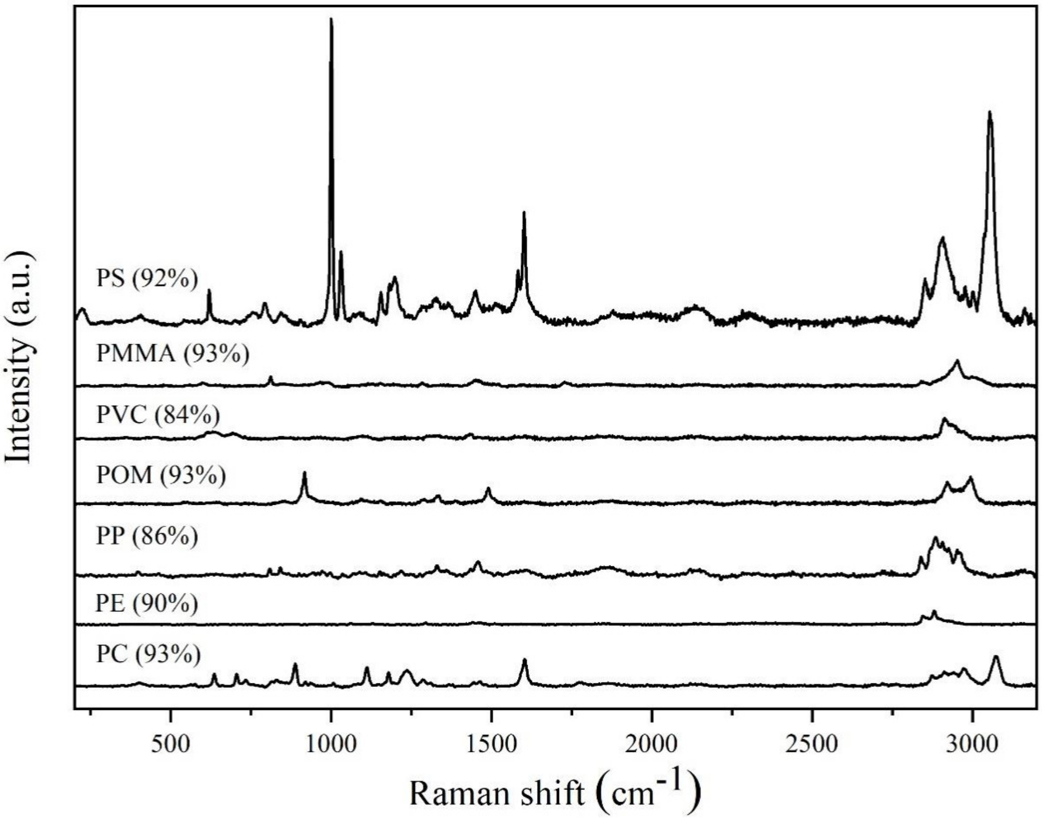

Figure S5. Partial Raman spectra of the detected MPs from water samples. PS – polystyrene, PMMA – poly(methyl methacrylate), PVC – polyvinylchloride, POM – polyformaldehyde, PP – polypropylene, PE – polyethylene, PC – polycarbonate. Percentage indicates match degree.

Table S1 Comparison of the abundance of MPs.

Location	Size range	Analysis methods	MPs abundance ($MPs \cdot L^{-1}$)			Main polymer type (total number)	MPs shape	References
			Raw water	Treated water	Tap water			
Czech Republic	>1 μm	FTIR, Raman	1473 ± 34<br>1812 ± 35<br>3605 ± 497	443 ± 10<br>338 ± 76<br>628 ± 28	-	PET, PP, PE (12)	fragment, fiber, spherical	[22]
Czech Republic	>0.2 μm	Raman	23 ± 2<br>1296 ± 35	14 ± 1<br>151 ± 4	-	PET, PVC, PP (13)	fragment, fiber	[23]
Bangladesh	>20 μm	ATR-FTIR	25.7 ± 9.8<br>26.0 ± 9.8<br>6.2 ± 1.6	0.3 ± 0.03<br>0.4 ± 0.01<br>0.05 ± 0.02	-	PP, PE, PET, PS (4)	fragment, fiber	[21]
Tehran, Iran	>1 μm	FTIR, Raman	2255 ± 383<br>1588 ± 313<br>1933 ± 381	1356 ± 264<br>1022 ± 259<br>1222 ± 288	-	PP, PET, PP (10)	fragment, fiber	[20]
Zahedan, Iran	>5 μm, >100 μm	FTIR, Raman	0.128 ± 0.022<br>0.078 ± 0.03	0.045 ± 0.01<br>0.020 ± 0.008	0.201 ± 0.113<br>0.206 ± 0.106	PP, PS (9)	fragment, fiber, film, foam	[34]
Busan, South Korea	>10 μm	FTIR	2.2 ± 1.3	0.02 ± 0.02	-	PP, PE, PET (10)	fragment, fiber	[37]
Catalonia, Spain	>20 μm	FTIR	0.96 ± 0.46	0.06 ± 0.04	-	PES, PP, PE (11)	fragment, fiber	[6]
Tianjin, China	>0.7 μm	FTIR	134.79	95.63	13.23	Nylon, PEST (7)	fragment, fiber, spherical	[29]
China	>1 μm	Raman	6614 ± 1132	930 ± 71	-	PE, PET, PP (>4)	fragment, fiber, spherical	[24]

Changsha, China	>1 um	FTIR, Raman	2753	351.9	343.5	PE, PP, PET (>7)	fragment, fiber, spherical	[25]
Jiaxing, China	>5 µm	Raman	3738.36 ± 461.29	695.66 ± 39.32	-	PP, PE, PVC (>7)	fragment, fiber	[10]
Haikou, China	>1 µm	Raman	0.8 ± 0.9<br>0.1 ± 0.7<br>0.7 ± 0.1	7.1 ± 1.6<br>6.7 ± 1.4<br>2.0 ± 0.4	1.4 ± 1.0<br>1.5 ± 1.8<br>0.8 ± 0.6	PP, PE, PVC (11)	fragment, fiber, spherical	This study

Table S2 Potential risk of different MPs in raw water.

Polymers	Abundance items/L	Chemical toxicity coefficient (Ti)	Potential single risk index (Ei)	Potential risk index (RI)
PP	0.11	1	0.02	13.46
PE	0.40	11	0.80	
POM	0.04	1500	12.15	
PS	0.09	30	0.49	

Table S3 Potential risk of different MPs in treated water.

Polymers	Abundance items/L	Chemical toxicity coefficient (Ti)	Potential single risk index (Ei)	Potential risk index (RI)
PP	2.22	1	0.41	370.26
PE	1.69	11	3.41	
POM	1.30	1500	357.80	
PC	0.04	1177	8.64	

Table S4 Potential risk of different MPs in tap water.

Polymers	Abundance items/L	Chemical toxicity coefficient (Ti)	Potential single risk index (Ei)	Potential risk index (RI)
PP	0.31	1	0.06	303.85
PE	0.64	11	1.30	
PVC	0.15	10551	291.89	
POM	0.02	1500	6.65	
PC	0.02	1177	3.57	
PS	0.02	30	0.09	
PET	0.01	4	0.01	
PMMA	0.00	1021	0.29	

Cellulose acetates, Polydimethylsiloxane, Unclear lacks toxicity data, therefore its hazard score cannot be determined.

Table S5 The EDI of MPs ingested by adults, children and infants via drinking water.

Location	C (particles·L^{-1})	IR ($L \cdot d^{-1}$)			BW (Kg)			EDI (MPs/kg/d)			References
		Adult	Children	Infant	Adult	Children	Infant	Adult	Children	Infant	
Zahedan, Iran	0.203	2-2.5	1-1.5	-	70	16	-	0.0002-0.0157	0.0004-0.0413	-	[30]
Beijing, China	49.67	1.85	1.12	0.60	60.6	27.25	9.65	2.68	3.61	5.47	[29]
Haikou, China	1.19							0.036	0.049	0.074	This work
